# Disturbance rejection model predictive control of lower limb rehabilitation exoskeleton

**DOI:** 10.1038/s41598-023-46885-4

**Published:** 2023-11-09

**Authors:** Xin Jin, Jia Guo

**Affiliations:** 1 School of Mechanical Engineering, Nanjing University of Science and Technology, Xiaolinwei Street No. 200, Nanjing, 210094 Jiangsu China; 2 School of Electrical and Computer Engineering, Georgia Institute of Technology, North Ave NW, Atlanta, Georgia 30332 USA

**Keywords:** Fracture repair, Biomedical engineering, Mechanical engineering

## Abstract

Nowadays, exoskeleton is broadly used in the rehabilitation training of many postoperative patients. However, the uncertainty and disturbances caused by different patients and system itself may lead to incompletely rehabilitation training as planned, or even unsafety. This paper addresses the control problem of a lower limb exoskeleton, in the spirit of the recent progress on model predictive control (MPC) and extended state observer (ESO). More precisely, our approach is based on the strategy that designing an ESO to estimate the total disturbance of the dynamics model and compensating it in the design of the MPC process. To accomplish this, we introduce the virtual control quantity to decouple the dynamics model of the system and summarize the human disturbances, unmeasured states and system non-linearity as the total disturbance of the model. By doing so, the uncertainty can be estimated by our designed ESO. Based on the moving horizontal optimization and feedback mechanism of MPC, the output prediction of the system can be more accurate since the uncertainty are effectively compensated. The virtual experiment results demonstrate that proposed controller significantly improves the control accuracy on lower limb rehabilitation exoskeleton with disturbances (improved by over 34$\%$), comparing with conventional MPC and fuzzy PID. As a result, our achievements will make contributions to better rehabilitation training for patients using rehabilitation exoskeletons.

## Introduction

In the past few decades, powered exoskeletons are widely used in medical rehabilitation, disaster relief and military technology^1–3^. In the field of rehabilitation engineering, exoskeleton research is dedicated to develop technology and systems for assisting patients with motor impairment. The integrated advantage of these devices, such as reaction rate, advanced control algorithms and human robot interaction, etc., could help the patients to perform necessary therapeutic training in daily life without help from the medical staff. With the demand of better user experience in recent year, these robots are required to have more ability of proactive behaviours, and planning patients’ motion in complex environments, which keeps anti-disturbance as an important issue as well as efficiency.

Traditional linear control methods (e.g. proportional integral derivative (PID) as a typical representative) has an important influence on control theory and engineering practice. But one of its disadvantages is that it is incapable to handle strong nonlinear disturbances or modeling disturbances. With the development of technology, many advanced nonlinear control techniques have been proposed to improve the tracking overall performance of the systems with disturbances and uncertainty, such as sliding mode control^4,5^, adaptive and robust control^6–8^, and neural network control^9^. These remarkable designs successfully solve the control problem of parameter uncertain systems and nonlinear disturbance systems and achieve better performances than the conventional linear controllers. However, most of the aforementioned control approaches adopt full-state feedback control strategy, which means all state information (i.e. displacement, velocity, and acceleration) are necessary. In practical perspective, only displacement information can be obtained relatively easily due to the potential structure limitations or power limitations in most exoskeleton systems. Besides, the measurements of velocity and acceleration usually contain noise factors, which will also degenerate the tracking performance of full-state based control methods. Therefore, introducing a feedback controller that only relay on position information becomes a natural progression.

As is known to all, MPC method can be regarded as an effective tool to deal with some uncertainties due to its mechanism of feedback correction and moving horizon optimization. Besides, it can tackle the complex nonlinear constraint flexibly. MPC can theoretically achieve the optimal control of the system more easily, which also drive the further development and wide application of MPC with its superior features. The basic idea of MPC^10–13^ is to deriving the output prediction equation from the identifiable dynamic model of the controlled system, build the objective function based on the prediction and control increment, and optimizing the quadratic objective function to generate the optimal control law of the current state. Initially, the application of MPC is constrained to the slow-varying systems (e.g. industrial fields such as oil, electricity and aviation) due to the limitation of computation ability. Besides, the sampling period of the discretization need to be long enough to ensure the complete execution of the control algorithm^14^. In latest decades, with the improvement of computing hardware, MPC has been utilized to fast systems such as autopilot system and robotic control.

Despite having these impressive merits, the tracking performance of MPC is still improvable. One of the problems is that the control effect relies on the accuracy of the model, which could be reduced by disturbances and uncertainty^15^ A possible way to solve this issue is to design a disturbance observer. As an interesting control topic, the disturbance observer concept has been presented as an effective tool to estimate the influence of disturbances on control by treating some terms of the dynamic model as disturbances^16^. The idea is further developed into various forms of observer-based controllers. For example,^17^ proposed an adaptive output-feedback controller based on fuzzy state observer for uncertain strict-feedback nonlinear systems,^18^ synthesized a nonlinear robust controller for hydraulic system control through back-stepping method and extended state observer (ESO), and^19^ studied the integration of MPC with the ESO to revise the prediction model, to name a few. Moreover, many outstanding accomplishments have additionally been achieved though ESO-based MPC in tracking control problem of quad-rotor helicopter with wind disturbances^20^ and in hydraulic systems with disturbances and uncertainties^21^, as well as in power converter systems under parametric uncertainties^22^. It is worth mentioning that these control studies can yield satisfactory performance and have rigorous theoretical properties. Consequently, it is expectable to exploit the ESO and MPC simultaneously for the control of power exoskeletons under disturbances, which encourages the motivation of this paper.

This paper proposed an MPC strategy for lower limb exoskeleton, inspired by the recent progress on linear ESO^18^ and model predictive control. The fundamental thought of our approach is that the quadratic objective function for solving the final control law is designed based on the uncertainties and disturbances, which are estimated by the designed ESO. The coupling non-linearity, unmeasured states, and disturbances from the wearers are all taken into consideration. Our contributions include the following aspects. (1) Introducing virtual variables into the dynamic model of the exoskeleton, which solves the decoupling problem of the nonlinear multi-input-multi-output (MIMO) system. (2) Integrating ESO with MPC to achieve tracking performance improvement of the exoskeleton under strong disturbances. (3) Proposing an ESO-based MPC controller that only depends on the position information of the exoskeleton, which improves the practicability. Theoretical analysis proves that the proposed controller can achieve a specified tracking performance. Comparative experiment results demonstrate that our design significantly outperforms the conventional MPC in tracking effectiveness and priority.

This paper is organized as follows: Section "Dynamic models" presents the dynamic model of the discussed exoskeleton. Section "Methods" presents the design of ESO, MPC controller and the proof of stability. Section "Experiment and results" presents the set up of virtual experiment and results. Section "Conclusions" is the conclusions.

## Dynamic models

**Figure 1:**
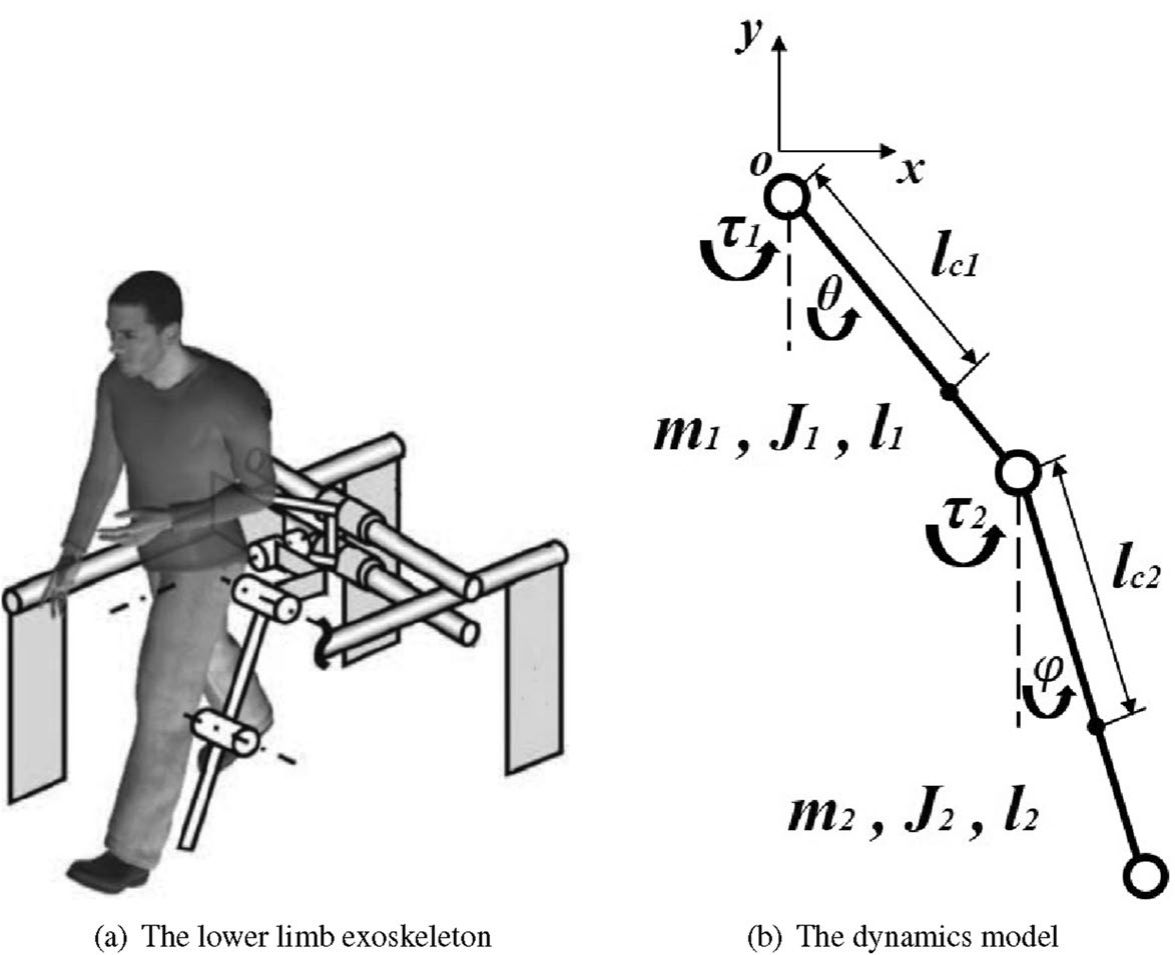
Illustrations of the lower limb exoskeleton (left) and the dynamics model in sagittal plane (right). Each limb of the exoskeleton is composed of two rotary joints (hip and knee) and two links (thigh and shank), and modeled as a double pendulum.

In this section, a two degrees of freedom (DoFs) lower limb exoskeleton is discussed, from which the dynamics model for the MPC controller is derived. Each limb of the exoskeleton is composed of two rotary joints and two links and is modeled as a double pendulum. The two joints are placed at the hip and knee of the wearer respectively, and the two links are fixed with thigh and shank respectively. The model of the lower limb exoskeleton is depicted in Fig. 1. It is worth noting that O point, the center of the waist, is relatively fixed in space or moving at a constant speed. This assumption is consistent with the practical application of rehabilitation exoskeletons, where the patient is relatively fixed by a bracket or sling and moves forward at a constant speed. The MPC control scheme is to make the exoskeleton taking a step following the set trajectory autonomously. To simplify the study, we only considered the swing action of one limb during a stepping cycle in sagittal plane (anterior-posterior direction). Thus, two DoFs of the motion are monitored, namely hip flexion angle $\theta$ and knee flexion angle $\varphi$.

The double pendulum can be considered as a potentially chaotic system, since a slight change of certain parameters in initial conditions can cause dramatic impacts on the subsequent motion. The objective of this study is to make the double links track the motion trajectory of the thigh and shank as closely as possible to ensure that the smooth and safe rehabilitation training of the patients, which means the tracking error under disturbances must be reduced to the minimum. Based on Second Lagrange equations, the dynamics of the exoskeleton can be described as,

$$\begin{aligned} \left\{ \begin{array}{c} \tau _1=\left( m_1 l_{c 1}^2+m_2 l_1^2+J_1\right) \ddot{\theta } +m_2 l_1 l_{c 2} \cos (\theta -\varphi ) \ddot{\varphi } +m_2 l_1 l_{c 2} \sin (\theta -\varphi ) \dot{\varphi }^2 \\ +\left( m_1 g l_{c 1}+m_2 g l_1\right) \sin \theta +\tau _t \\ \tau _2=m_2 l_1 l_{c 2} \cos (\theta -\varphi ) \ddot{\theta } +\left( m_2 l_{c 2}^2+J_2\right) \ddot{\varphi }-m_2 l_1 l_{c 2} \sin (\theta -\varphi ) \dot{\theta }^2 \\ +m_2 g l_{c 2} \sin \varphi +\tau _s \end{array}\right. \end{aligned}$$

where $m_{1}, l_{1}$ and $J_{1}$ represent the mass, the moment of inertia and the length of the thigh, respectively. $m_{2}, J_{2}$ and $l_{2}$ represent the mass, the moment of inertia and the length of the shank, respectively. $\dot{\theta }$ and $\ddot{\theta }, \dot{\varphi }$ and $\ddot{\varphi }$ are the angular velocity and angular acceleration of hip joint and knee joint, respectively. $l_{c 1}$ and $l_{c 2}$ represent the distances from the joints to the mass centers of the two legs. $\tau _{1}$ and $\tau _{2}$ are the torques exerted respectively on the hip joint and knee joint. $\tau _{t}$ and $\tau _{s}$ are the unknown torques generated by human-machine interaction.

In practice, it is reasonable to assume that the unknown control torque $\tau _{t}$ and $\tau _{s}$ are continuously differentiable. Define the state variable vector as $x=\left[ x_{1}, x_{2}, x_{3}, x_{4}\right] ^{T}=$ $[\theta , \dot{\theta }, \varphi , \dot{\varphi }]^{T}$, the control input vector as $u=\left[ u_{1}, u_{2}\right] ^{T}=\left[ \tau _{1}, \tau _{2}\right] ^{T}$. Therefore, based on the dynamics model of the system, the exoskeleton system is presented in state-space form as,

1 $$\begin{aligned} \left\{ \begin{array}{c} \dot{x}=M^{-1} \cdot f^M\left( x, u, \tau _d\right) =B_0 \cdot u+d\left( x, \tau _d\right) \\ y=C x \end{array}\right. \end{aligned}$$

where $$B_{0}=\gamma \left[ \begin{array}{cc}0 &{} 0 \\ -\left( m_{2} l_{c 2}^{2}+J_{2}\right) &{} m_{2} l_{1} l_{c 2} \cos (\theta -\varphi ) \\ 0 &{} 0 \\ m_{2} l_{1} l_{c 2} \cos (\theta -\varphi ) &{} -\left( m_{1} l_{c 1}^{2}+m_{2} l_{1}^{2}+J_{1}\right) \end{array}\right] $$, $$d\left( x, \tau _{d}\right) =\left[ \begin{array}{c}\dot{\theta } \\ d_{I} \\ \dot{\varphi } \\ d_{I I}\end{array}\right] $$, $$C=\left[ \begin{array}{llll}1 &{} 0 &{} 0 &{} 0 \\ 0 &{} 0 &{} 0 &{} 0 \\ 0 &{} 0 &{} 1 &{} 0 \\ 0 &{} 0 &{} 0 &{} 0\end{array}\right] $$,

$$M=\begin{bmatrix} 1 &{} 0 &{} 0 &{} 0 \\ 0 &{} m_{1}l_{c1}^{2}+m_{2}l_{1}^{2}+J_{1} &{} 0 &{} m_{2}l_{1}l_{c2}cos\left( \theta -\varphi \right) \\ 0 &{} 0 &{} 1 &{} 0 \\ 0 &{} m_{2}l_{1}l_{c2}cos\left( \theta -\varphi \right) &{} 0 &{} m_{2}l_{c2}^{2}+J_{2} \\ \end{bmatrix}$$.

where $d\left( x, \tau _{d}\right)$ are considered as the total disturbance of the system, which are caused by the human-machine interaction, nonlinear parameters deviations and so on. $\gamma$ is the coefficient. They are determined as follows,

$$\begin{aligned} d_{I}&=\gamma \left( m_{2} l_{c 2}^{2}+J_{2}\right) \cdot \left( m_{2} l_{1} l_{c 2} \sin (\theta -\varphi ) \dot{\varphi }^{2}+\left( m_{1} g l_{c 1}+m_{2} g l_{1}\right) \sin \theta -\tau _{t}\right) \\&\quad +\gamma m_{2} l_{1} l_{c 2} \cos (\theta -\varphi ) \cdot \left( m_{2} l_{1} l_{c 2} \sin (\theta -\varphi ) \dot{\theta }^{2}-m_{2} g l_{c 2} \sin \varphi -\tau _{s}\right) ,\\ d_{I I}&=-\gamma m_{2} l_{1} l_{c 2} \cos (\theta -\varphi ) \cdot \left( m_{2} l_{1} l_{c 2} \sin (\theta -\varphi ) \dot{\varphi }^{2}+\left( m_{1} g l_{c 1}+m_{2} g l_{1}\right) \sin \theta -\tau _{t}\right) \\&\quad -\gamma \left( m_{1} l_{c 1}^{2} +m_{2} l_{1}^{2}+J_{1}\right) \left( m_{2} l_{1} l_{c 2} \sin (\theta -\varphi ) \dot{\theta }^{2}-m_{2} g l_{c 2} \sin \varphi -\tau _{s}\right) , \\ \gamma&=\frac{1}{m_{2}^{2} l_{1}^{2} l_{c 2}^{2} \cos ^{2}(\theta -\varphi )-\left( m_{2} l_{c 2}^{2} +J_{2}\right) \left( m_{1} l_{c 1}^{2}+m_{2} l_{1}^{2}+J_{1}\right) }. \end{aligned}$$

It is worth mention that in this practical work, we focused on applications and used the continuous dynamical model to model the system and discretized it for more convenient use, and then, all the stability analysis is made upon the discretized version.

## Methods

### Model analysis

In this section, the physical parameters of exoskeleton system (i.e. $m, l, J, \theta , \varphi$ ) are utilized in the design of ESO and MPC controller, based on the MPC theory. Obviously, it can be seen from model (1) that the system is always subjected to the parametric uncertainties and disturbances (i.e. the uncertain time-varying parameters *x* and $\tau _{d}$ in $d\left( x, \tau _{d}\right)$). Accordingly, we treat the total uncertainty of the model in the state-space equation as the main disturbances, which would be estimated by the designed observer and then compensated in the MPC controller to enhance the tracking performance and robustness of the system under disturbances. In order to accomplish this task, we must first solve the problem of system decoupling.

For strong coupling systems, decoupling control is an effective solution. The decoupling process^23^ is realized by solving the inverse matrix using the matrix decomposition method. This method is not only simple, but also capable to deal with the time-varying matrix, so it can achieve the effect of real-time control. In addition, this method has relatively sufficient mathematical guarantee. By introducing virtual control variables: $$u^{D}=D \cdot u=\left[ \begin{array}{cc}-\left( m_{2} l_{c 2}^{2}+J_{2}\right) &{} m_{2} l_{1} l_{c 2} \cos (\theta -\varphi ) \\ m_{2} l_{1} l_{c 2} \cos (\theta -\varphi ) &{} -\left( m_{1} l_{c 1}^{2}+m_{2} l_{1}^{2}+J_{1}\right) \end{array}\right] \cdot u$$, the input-output relationships of link *i* of the exoskeleton system can be written as

2 $$\begin{aligned} \left\{ \begin{array}{c} \ddot{x}_{i}=\gamma \cdot u_{i}^{D}+d_{i}(x, t) \\ y_{i}=x_{i}\end{array} \quad i=I, II\right. \end{aligned}$$

This means the input on link *i* is $u_{i}^{D}$ and its output is $y_{i}=x_{i}$, so there is a single input-single output relationship between the virtual control variable $u_{i}^{D}$ and the controlled output $y_{i}$ of each link. That is, the controlled output $y_{i}$ of link *i* and the virtual control variable $u_{i}^{D}$ are completely decoupled. Therefore, the decoupling control of multivariable system can be realized by embedding *n* controllers in parallel between the control vector $u^{D}$ and the output vector *y*. Meanwhile, the actual control quantity *u* can be determined by the formula of the virtual control quantity $u^{D}$

3 $$\begin{aligned} u=D^{-1} \cdot u^{D} \end{aligned}$$

Therefore, the original state-space Eq. (1) of the system can be written as,

4 $$\begin{aligned} \dot{x}=A \cdot x+B \cdot u^{D}+d \end{aligned}$$

where $$A=\left[ \begin{array}{llll}0 &{} 1 &{} 0 &{} 0 \\ 0 &{} 0 &{} 0 &{} 0 \\ 0 &{} 0 &{} 0 &{} 1 \\ 0 &{} 0 &{} 0 &{} 0\end{array}\right] $$,    $$B=\left[ \begin{array}{ll}0 &{} 0 \\ \gamma &{} 0 \\ 0 &{} 0 \\ 0 &{} \gamma \end{array}\right] $$,    $$d=\left[ 0, d_{I}, 0, d_{I I}\right] ^{T}$$.

Let the sampling time of the system be $T_{s}$. Notice that the sampling time for the system has to be short enough to meet the performance requirements of the exoskeleton system. Applying the forward Euler discrete method to (4), the discrete time model of the system is given by,

5 $$\begin{aligned} \left\{ \begin{array}{c} x(k+1)=\tilde{A} \cdot x(k)+\tilde{B} \cdot u^D(k)+I_{T_S} \cdot \hat{d}(k) \\ y(k)=C \cdot x(k) \end{array}\right. \end{aligned}$$

where $$\tilde{A}=\left( I+T_{S} \cdot A\right) $$, $$\tilde{B}=T_{S} \cdot B$$, $$I_{T_{S}}=T_{S} \cdot I$$.

For the design of MPC controller and ESO, the following assumptions are made in this paper.

#### Assumption 1

The time-varying non-linear disturbances $d_{I}$ and $d_{I I}$ in (1) are both bounded and satisfy

$$\begin{aligned} \left| d_{I}\right|<\delta _{1}, \left| d_{I I}\right|<\delta _{2}, \left| \dot{d}_{I}\right|<\delta _{3}, \left| \dot{d}_{I I}\right| <\delta _{4}. \end{aligned}$$

where $\delta _{1}, \delta _{2}, \delta _{3}$ and $\delta _{4}$ are positive constant.

#### Assumption 2

According to the MPC theory, let the prediction horizon as $N_{p}$ and the control horizon of the system as $N_{c}\left( N_{c} \le N_{p}\right)$. The state prediction equation and output prediction equation can be derived from the state-space equation based on the following assumptions,

$$\begin{aligned}{}&\Delta u(k+i)=0 \quad if i=N_{c}, N_{c}+1, \cdots , N_{p}-1 \\&\Delta d_{I}(k+i)=\Delta d_{I I}(k+i)=0 \quad if i=1,2, \cdots , N_{p}-1 \\&\Delta \gamma =0 \quad if i=1,2, \cdots , N_{p}-1 \end{aligned}$$

where $\Delta u, \Delta d$ and $\Delta \gamma$ denote the increment of control law *u*, system disturbance *d* and coefficient $\gamma$ respectively.

### ESO design

The objective of designing ESO is to estimate the total model disturbances in real time. Accordingly, we first extend the uncertainties $d_{I}$ and $d_{I I}$ as additional state variables, and let $h_{1}$ and $h_{2}$ represent the time derivative of $d_{I}$ and $d_{I I}$ respectively (i.e. $h_{I}=\dot{d}_{I}, h_{I I}=\dot{d}_{I I}$ ). The extended states are defined as $x_{e}=$ $\left[ x_{e 1}, x_{e 2}, x_{e 3}, x_{e 4}, x_{e 5}, x_{e 6}\right] ^{T}=\left[ \theta , \dot{\theta }, d_{I}, \varphi , \dot{\varphi }, d_{I I}\right] ^{T}$. From (5), the extended state-space equation of the system is expressed as follows,

6 $$\begin{aligned} \left\{ \begin{array}{c} \dot{x}_{e}=A_{e} \cdot x_{e}+B_{e} \cdot u^{D}+H_{e} \\ y(k)=C_{e} \cdot x_{e}(k) \end{array}\right. \end{aligned}$$

where $$A_{e}=\left[ \begin{array}{llllll}0 &{} 1 &{} 0 &{} 0 &{} 0 &{} 0 \\ 0 &{} 0 &{} 1 &{} 0 &{} 0 &{} 0 \\ 0 &{} 0 &{} 0 &{} 0 &{} 0 &{} 0 \\ 0 &{} 0 &{} 0 &{} 0 &{} 1 &{} 0 \\ 0 &{} 0 &{} 0 &{} 0 &{} 0 &{} 1 \\ 0 &{} 0 &{} 0 &{} 0 &{} 0 &{} 0\end{array}\right] , B_{e}=\left[ \begin{array}{ll}0 &{} 0 \\ \gamma &{} 0 \\ 0 &{} 0 \\ 0 &{} 0 \\ 0 &{} \gamma \\ 0 &{} 0\end{array}\right] , H_{e}=\left[ \begin{array}{c}0 \\ 0 \\ h_{I} \\ 0 \\ 0 \\ h_{I I}\end{array}\right] $$.

Let *z* denotes the estimation of $x_{e}$. From the extended system model (6), a linear form ESO^18^ can be designed as follows,

7 $$\begin{aligned} \dot{z}=A_{e} \cdot z+B_{e} \cdot u^{D}+L \cdot (y-\hat{y}) \end{aligned}$$

where $L=\left[ 3 \omega _{o}, 3 \omega _{o}^{2}, \omega _{o}^{3}, 3 \omega _{o}, 3 \omega _{o}^{2}, \omega _{o}^{3}\right] ^{T}$ represents the observer gain and $\omega _{o}>0$ are considered as the bandwidth of the observers.

To analyze the designed ESO^24^, the characteristic polynomial in (7) can be inferred to

8 $$\begin{aligned} \lambda (s)=\left( s+\omega _{o}\right) ^{3} \end{aligned}$$

Let $\tilde{x}_{e}$ represents the estimated error (i.e. $\tilde{x}_{e}=x_{e}-z$). From (6) and (7), the estimation error can be shown as follows,

9 $$\begin{aligned} \dot{\tilde{x}}_{e}=\left[ \begin{array}{cccccc} -3 \omega _{o} &{} 1 &{} 0 &{} 0 &{} 0 &{} 0 \\ -3 \omega _{o}^{2} &{} 0 &{} 1 &{} 0 &{} 0 &{} 0 \\ -\omega _{o}^{3} &{} 0 &{} 0 &{} 0 &{} 0 &{} 0 \\ 0 &{} 0 &{} 0 &{} -3 \omega _{o} &{} 1 &{} 0 \\ 0 &{} 0 &{} 0 &{} -3 \omega _{o}^{2} &{} 0 &{} 1 \\ 0 &{} 0 &{} 0 &{} -\omega _{o}^{3} &{} 0 &{} 0 \end{array}\right] \cdot \tilde{x}_{e}+H_{e} \end{aligned}$$

Define $\varepsilon =\textrm{diag}\left( 1, \omega _{o}^{-1}, \omega _{o}^{-2}, 1, \omega _{o}^{-1}, \omega _{o}^{-2}\right) \cdot \tilde{x}_{e}$, the estimation error can be rewritten as

10 $$\begin{aligned} \dot{\varepsilon }=\omega _{o} A_{\varepsilon } \cdot \varepsilon +M_e \frac{H_{e}}{\omega _{o}^{2}} \end{aligned}$$

where $M_e=[0,0,1,0,0,1]^{T}$ and $A_{\varepsilon }$ is the Hurwitz matrix that can be inferred from (9).

#### Lemma 1

Reference^24^ Assuming that $H_{e}$ is bounded, then the estimated states are always bounded. There exist a constant $\sigma _{i}>0$, some positive integer *c* and a finite time $T_{1}>0$, that satisfy the following equation,

$$\begin{aligned} \left| \tilde{x}_{ei} \right| \le \sigma _{i}, \quad \sigma _{i}=O\left( \frac{1}{\omega _{o}^{c}}\right) , \quad i=1,2, \cdots , 6 \quad \forall t \ge T_{1} \end{aligned}$$

From Lemma 1 and (10), it can be inferred that the designed ESO in (7) is stable. In addition, the estimation error $\tilde{x}_{e}$ of system uncertainties can be made arbitrarily small by increasing the bandwidth $\omega _{o}$, according to the primary analysis^24,25^.

Therefore, the estimated values of total disturbances $d_{I}$ and $d_{I I}$ can be obtained from the observed value $z_{3}$ and $z_{6}$ (i.e. $\hat{d}_{I}=z_{3}, \hat{d}_{I I}=z_{6}$). The discrete time model of the ESO is given by,

11 $$\begin{aligned} \left\{ \begin{array}{c} z(k+1)=\tilde{A}_{e} \cdot z(k)+\tilde{B}_{e} \cdot u^{D}(k) +\tilde{L} \cdot (y(k)-\hat{y}(k)) \\ \hat{y}(k)=C_{e} \cdot z(k) \end{array}\right. \end{aligned}$$

where $$\tilde{A}_{e}=I+T_{s} \cdot A_{e}, \tilde{B}_{e}=T_{s} \cdot B_{e}, \tilde{L}=T_{s} \cdot L$$.

### MPC controller design

By subtracting the states of two adjacent sampling instants, the incremental form model of the system can be derived from (5). It is expressed as,

12 $$\begin{aligned} \left\{ \begin{array}{c} \Delta x(k+1)=\tilde{A} \cdot \Delta x(k)+\tilde{B} \cdot \Delta u^{D}(k)+I_{T_{S}} \cdot \Delta \hat{d}(k) \\ y(k)=C \cdot \Delta x(k)+y(k-1) \end{array}\right. \end{aligned}$$

where $\Delta x(k)=x(k)-x(k-1)$ is the state increment, $\Delta u^{D}(k)=u^{D}(k)-u^{D}(k-$ 1) is the control increment, $\Delta \hat{d}(k)=\hat{d}(k)-\hat{d}(k-1)$ is the disturbance increment. Taking the current time step *k* and the current system state *x*(*k*) as the initial conditions. The state increment prediction $\Delta x(k+1)$ at time step $k+1$ will be represented as $\Delta x(k+1 \mid k)$. The predictive system output $y(k+1)$ at time step $k+1$ will be represented as $y(k+1 \mid k)$. Assuming that the prediction time domain is $N_{p}$ and the control time domain is $N_{c}$. Based on Assumption 2 and (12), the state prediction equation can be conducted as follows,

13 $$\begin{aligned} \Delta x(k+1 \mid k)&= \tilde{A} \cdot \Delta x(k)+\tilde{B} \cdot \Delta u^{D}(k)+I_{T_{s}} \cdot \Delta \hat{d}(k), \nonumber \\ \Delta x(k+2 \mid k)&= \tilde{A}^{2} \cdot \Delta x(k)+\tilde{A} \cdot \tilde{B} \cdot \Delta u^{D}(k)+\tilde{B} \cdot \Delta u^{D}(k+1)+\tilde{A} \cdot I_{T_{s}} \cdot \Delta \hat{d}(k), \nonumber \\&\qquad \cdots \nonumber \\ \Delta x\left( k+N_{p} \mid k\right)&= \tilde{A}^{N_{p}} \cdot \Delta x(k)+\tilde{A}^{N_{p}-1} \cdot \tilde{B} \cdot \Delta u^{D}(k)+\tilde{A}^{N_{p}-2} \cdot \tilde{B} \cdot \Delta u^{D}(k+1) \nonumber \\&\quad +\cdots +\tilde{A}^{N_{p}-N_{c}} \cdot \tilde{B} \cdot \Delta u^{D}\left( k+N_{c}-1\right) +\tilde{A}^{N_{p}-1} \cdot I_{T_{s}} \cdot \Delta \hat{d}(k) \end{aligned}$$

Additionally, the output prediction equation can be derived based on (12) and (13). It is expressed as follows,

14 $$\begin{aligned} y(k+1 \mid k)&= C \cdot \tilde{A} \cdot \Delta x(k) +C \cdot \tilde{B} \cdot \Delta u^{D}(k)+C \cdot I_{T_{s}} \cdot \Delta \hat{d}(k)+y(k), \nonumber \\&\qquad \cdots \nonumber \\ y\left( k+N_{c} \mid k\right)&= \sum _{i=1}^{N_{c}} C \cdot \tilde{A}^{i} \cdot \Delta x(k)+\sum _{i=1}^{N_{c}} C \cdot \tilde{A}^{i-1} \cdot \tilde{B} \cdot \Delta u^{D}(k) +\sum _{i=1}^{N_{c}-1} C \cdot \tilde{A}^{i-1} \cdot \tilde{B} \nonumber \\&\quad \cdot \Delta u^{D}(k+1)+\cdots +C \cdot \tilde{B} \cdot \Delta u^{D}\left( k+N_{c}-1\right) \nonumber \\&\quad + \sum _{i=1}^{N_{c}} C \cdot \tilde{A}^{i-1} \cdot I_{T_{S}} \cdot \Delta \hat{d}(k)+y(k), \nonumber \\ y\left( k+N_{p} \mid k\right)&= \sum _{i=1}^{N_{p}} C \cdot \tilde{A}^{i} \cdot \Delta x(k)+\sum _{i=1}^{N_{p}} C \cdot \tilde{A}^{i-1} \cdot \tilde{B} \cdot \Delta u^{D}(k) +\sum _{i=1}^{N_{p}-1} C \cdot \tilde{A}^{i-1} \nonumber \\&\quad \cdot \tilde{B} \cdot \Delta u^{D}(k+1)+\cdots +\sum _{i=1}^{N_{p}-N_{c}+1} C \cdot \tilde{A}^{i-1} \cdot \tilde{B} \cdot \Delta u^{D}\left( k+N_{c}-1\right) \nonumber \\&\quad +\sum _{i=1}^{N_{p}} C \cdot \tilde{A}^{i-1} \cdot I_{T_{s}} \cdot \Delta \hat{d}(k)+y(k) \end{aligned}$$

Define *Y*(*k*) and $\Delta U(k)$ as follows,

$$\begin{aligned} Y(k+1 \mid k)&=\left[ y(k+1 \mid k), y(k+2 \mid k), \cdots , y \left( k+N_{p} \mid k\right) \right] ^{T}\\ \Delta U^{D}(k)&=\left[ \Delta u^{D}(k), \Delta u^{D}(k+1), \cdots , \Delta u^{D}\left( k+N_{c}-1\right) \right] ^{T} \end{aligned}$$

Therefore, the output prediction equation (14) can be reformed as,

15 $$\begin{aligned} Y(k+1 \mid k)=H_{x} \Delta x(k)+H_{I} y(k)+H_{d} \Delta \hat{d}(k)+H_{u} \Delta U^{D}(k) \end{aligned}$$

where the matrices of $H_{x}, H_{I}, H_{d}$ and $H_{u}$ are determined by the matrices of $\tilde{A}, \tilde{B}$, $I_{T_{S}}$ and *C* in (14).

The estimated error in (15) has been neglected because it can be made small enough by increasing the ESO gains. The effect of the estimation error will be analyzed in the next subsection. The objective function of MPC is designed to reflect the control and tracking performance of the controlled exoskeleton system. And the optimal control sequence can be obtained by optimizing the quadratic objective function, in order to track the desired trajectory. The objective function of the controlled exoskeleton system is designed as follows,

16 $$\begin{aligned} J=\left\| R \cdot \left( Y(k+1 \mid k)-X_{r}(k+1)\right) \right\| ^{2} +\left\| Q \cdot \Delta U^{D}(k)\right\| ^{2} \end{aligned}$$

where the first term to the right of the equal sign is the terminal constraint, and the second term is the control law increment constraint (dynamic constraint). Where *R* and *Q* in (16) are the diagonal weight matrices of tracking error and control increment, respectively. In addition, $X_{r}(k+1)$ is an $N_{p}$-dimensional vector, which represents the reference trajectory. Substituting (15) to (16), the optimal control sequence solved can be expressed as,

17 $$\begin{aligned} \Delta U^{D}=\left( H_{u}^{T} R^{T} R H_{u}+Q^{T} Q\right) ^{-1} \cdot H_{u}^{T} R^{T} R \cdot E_{p}(k+1 \mid k) \end{aligned}$$

where $E_{p}(k+1 \mid k)=X_{r}(k+1)-H_{x} \Delta x(k)-H_{I} y(k)-H_{d} \Delta \hat{d}(k)$ can be calculated based on the reference trajectory and system outputs in real-time.

According to the moving horizontal optimization mechanism of MPC controller, only the first term of the optimized control sequence is input to the controlled system during each sampling period, and this optimization process will be repeated in the next sampling period. The optimal control law of MPC can be described by,

18 $$\begin{aligned} \Delta u^{D}(k)&=K_{m p c} \cdot E_{p}(k+1 \mid k) \nonumber \\ u^{D}(k)&=u^{D}(k-1)+\Delta u^{D}(k) \end{aligned}$$

where $K_{m p c}=[1,0, \cdots , 0]_{1 \times N_{c}}\left( H_{u}^{T} R^{T} R H_{u}+Q^{T} Q\right) ^{-1} \cdot H_{u}^{T} R^{T} R$ can be regarded as the predictive control gain. It is not hard to notice that the $K_{m p c}$ can be pre-calculated offline, which will reduce computing load of the processor.

### Stability proof

The stability of the proposed ESO-based MPC controller is analyzed in this subsection, based on the stability proof of Lyapunov theoretical framework. First, substituting $y(k)=C \cdot \Delta x(k)+y(k-1)$ to (17), it can be rewritten as,

19 $$\begin{aligned} \Delta u^{D}(k)&= K_{m p c} X_{r}(k+1)-K_{m p c} H_{x} \Delta x(k)-K_{m p c} H_{d} \Delta \hat{d}(k)-K_{m p c} H_{I} y(k) \nonumber \\&= K_{m p c} X_{r}(k+1)-K_{m p c}\left( H_{x}+H_{I} C\right) \Delta x(k)-K_{m p c} H_{d} \Delta \hat{d}(k) \nonumber \\&\quad -K_{m p c} H_{I} y(k-1) \end{aligned}$$

Substituting (19) to (12), the incremental model of the discrete system with disturbances can be written as follows,

20 $$\begin{aligned} \Delta x(k+1)&= \tilde{A} \cdot \Delta x(k)+\tilde{B} \cdot \Delta u^{D}(k)+I_{T_{s}} \cdot \Delta \hat{d}(k) \nonumber \\&= \left[ \tilde{A}-K_{m p c} \tilde{B}\left( H_{x}+H_{I} C\right) \right] \cdot \Delta x(k)+K_{m p c} \tilde{B} \cdot X_{r}(k+1) \nonumber \\&\quad +\left( I_{T_{s}}-K_{m p c} \tilde{B} H_{d}\right) \cdot \Delta \hat{d}(k)-K_{m p c} \tilde{B} H_{I} \cdot y(k-1) \end{aligned}$$

Similarly, substituting (19), $y(k)=C \cdot \Delta x(k)+y(k-1)$ and $\hat{y}(k)=C_{e} \cdot z(k)$ to (7), the incremental model of the discrete ESO can be derived as follows,

21 $$\begin{aligned} \Delta z(k+1)&= \tilde{A}_{e} \cdot z(k)+\tilde{B}_{e} \cdot u^{D}(k)+\tilde{L} \cdot (y(k)-\hat{y}(k))=\left[ \tilde{L} C-K_{m p c} \tilde{B}_{e}  \left( H_{x}+ H_{I} C\right) \right] \cdot \Delta x(k)+\left( \tilde{A}_{e}-\tilde{L} C_{e}\right) \cdot \Delta z(k)+K_{m p c} \tilde{B}_{e} \cdot X_{r}(k+1) \nonumber \\&\quad -K_{m p c} \tilde{B}_{e} H_{d} \cdot \Delta \hat{d}(k)+\left( \tilde{L}-K_{m p c} \tilde{B}_{e} H_{I}\right) \cdot y(k-1) \end{aligned}$$

Based on (20) and (21), the state increment of the closed-loop system is defined as,

$$\begin{aligned} \Delta x_{l}=\left[ \Delta x(k), \Delta z(k)\right] ^{T} \end{aligned}$$

Consequently, the closed-loop system of ESO-based MPC can be described as,

22 $$\begin{aligned} \Delta x_{l}(k+1)&= A_{l} \Delta x_{l}(k)+K_{m p c} \tilde{B}_{e} X_{r}(k+1)+\left[ \begin{array}{l} I_{T_{s}}-K_{m p c} \tilde{B} H_{d} \\ -K_{m p c} \tilde{B}_{e} H_{d} \end{array} \right] \Delta \hat{d}(k) \nonumber \\&\quad - \left[ \begin{array}{c} K_{m p c} \tilde{B} H_{I} \\ \tilde{L}-K_{m p c} \tilde{B}_{e} H_{I} \end{array}\right] y(k-1). \end{aligned}$$

where $$A_{l}=\left[ \begin{array}{ll}\tilde{A}-K_{m p c} \tilde{B}\left( H_{x}+H_{I} C\right) &{} 0 \\ \tilde{L} C-K_{m p c} \tilde{B}_{e}\left( H_{x}+H_{I} C\right) &{} \tilde{A}_{e}-\tilde{L} C_{e}\end{array}\right] $$.

The closed-loop system can obtain nominal asymptotic stability if the total eigenvalue of the matrix $A_{l}$ is within the unit circle^26^.

#### Remark 1

The parameters adjustment of the proposed controller and observer is discussed. For the MPC controller, the matrices *R* and *Q* in the objective function reflect the design expectation of the tracking performance and control increment. The matrix *R* is the weighting factor matrix of tracking error. The larger the matrix *R*, the closer the theoretical system output is to the reference trajectory. The matrix *Q* is the weighting factor matrix of control increment. The larger the matrix *Q*, the smaller the control quantity of the system. Meanwhile, the matrices of *R* and *Q* are also crucial to the stability of the closed-loop system. In practice, the overlarge matrix *R* may cause system instability due to the system state chattering. Therefore, in the process of matrix parameters adjustment, the matrix *R* is first adjusted based on the performance of tracking error, and then the matrix *Q* is adjusted to meet the limitation of the control increment and alleviate the control input chattering.

#### Remark 2

Another set of important parameters of the MPC controller is the prediction horizon $N_{p}$ and the control horizon $N_{c}$. Ideally, $N_{p}$ should be long enough to predict the system dynamic response as long as possible. However, it is unrealistic to predict the whole process due to the limitation of computational cost and time. Hence, a compromise scheme is usually adopted, which is taking the lower limit of the prediction horizon length. It is obvious that the change of $\Delta u$ at time step *k* will have a direct effect on the control input *u* at the next time step $k+1$, and then have a long-term effect on the system response of acceleration $\ddot{y}$ at the time step $k+2$, on the velocity $\dot{y}$ at the time step $k+3$ and on the displacement *y* at the time step $k+4$. Therefore, we choose a minimum value of the prediction horizon $N_{p}=5$ in the implementation. Apparently, the length of the control horizon $N_{c}$ should be less than the prediction horizon. $N_{c}$ represents the number of steps which the input control quantity is seen as constant when predicting the future output of the system^27^. Similarly, to reduce the calculation time and amount of the optimization process, we choose $N_{c}=2$ in this paper.

#### Remark 3

$\omega _{o}$ is considered as the bandwidth of the designed ESO. Theoretically, by increasing the bandwidth $\omega _{o}$, the estimation error of the total disturbance and state can be made arbitrarily small^28^. But overlarge $\omega _{o}$ means increasing the sensitivity of the ESO to the noise, which may cause the chattering of the estimation because of the amplification of the measurement noise, etc. The adjustment process of $\omega _{0}$ will be started from a small value and gradually increased to improve the performance of observation and the overall tracking performance until reaching a satisfied level in implementation.

## Experiment and results

In this section, we explore the tracking performance of the proposed ESO-based MPC controller when applied to the task of exoskeleton motion control. Through comparing with the conventional MPC strategy, we attempt to demonstrate the advantages of the proposed controller in tracking accuracy with disturbances.

### Setup and evaluation

**Figure 2:**
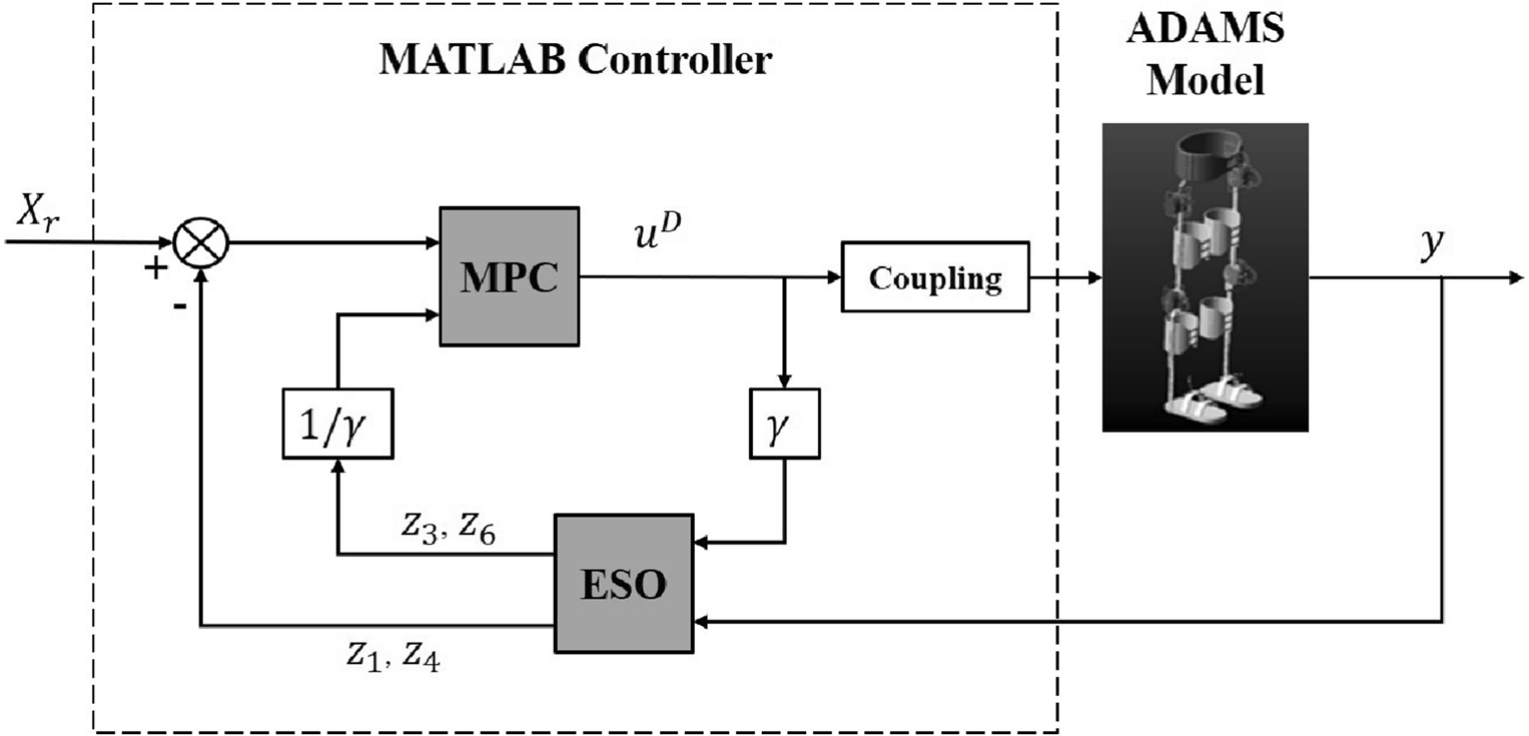
Control flow diagram of the co-simulation model. The deviation between the ESO estimated position and the reference position, combining with the total disturbance also obtained by ESO estimation, is substituted into the MPC controller to obtain the optimal control torque. The control quantity is input into the exoskeleton dynamics model to obtain the actual position, which is feed back to the controller to form a closed loop control system.

A co-simulation model of the exoskeleton system has been built up, as shown in Fig. 2, in order to test and verify the tracking performance of the proposed controller. We firstly model the existing lower limb exoskeleton product designed by our cooperative team^29^ in computer via ADAMS. Considering the calculation cost, some non-critical parts of the exoskeleton is simplified, such as screws, rubber pads, straps, etc. Besides, all joints of exoskeleton (i.e. hip, knee, ankle) are setup with CONTACT connections to simulate the real status of the system. For the power supply problem, we replace the actual driving motors (CARTRIDGE DDR Motor C043A and C053A, Kollmorgen, USA) with an ideal virtual driving force, and limit the control force increment in MPC controller based on the physical characteristics of the motor $(-50 N \le u_{1} \le +50 N,-25 N \le u_{2} \le +25 N)$. By doing so, we avoid discuss the power and control characteristics of the motor in co-simulation.

**Figure 3:**
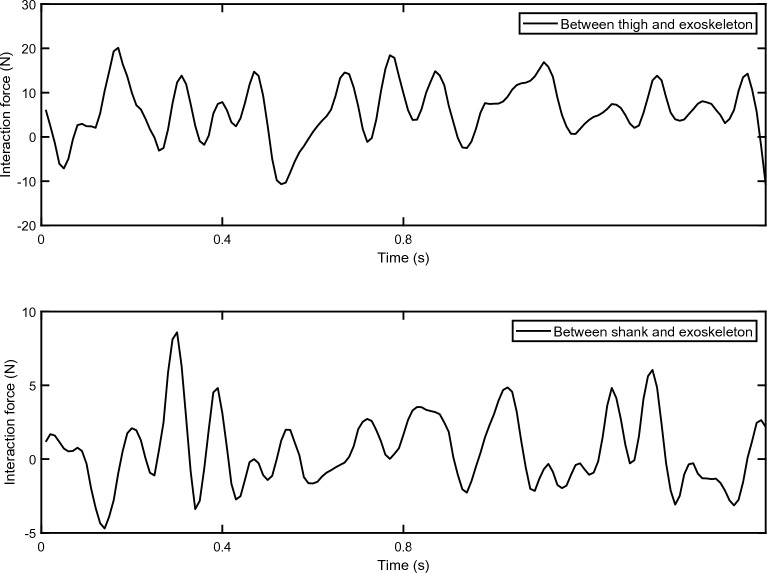
The interaction forces between human body and the exoskeleton. The upper sub-figure is the interaction force between thigh and the exoskeleton. The lower sub-figure is the interaction force between shank and the exoskeleton.

Though previous experiments, the interaction forces between human body and the exoskeleton has been measured, as shown in Fig. 3. It is considered as external disturbances and applied to the corresponding position of the model. The reference trajectory of the human lower limbs is given in the work of other researchers^30^. Based on the composition of exoskeleton system, the nominal values of each component parameters are listed in Table 1. The control strategy, which consists of ESO, MPC controller, software interaction and data collection, is realized via MATLAB Simulink. The sampling time of the control system is set as $T_{s}=0.01 s$.

**Table 1:** Parameters of the exoskeleton system.

Parameters	Physical meaning	Value
$m_{1} / \textrm{kg}$	Mass of thigh	3.71
$m_{2} / \textrm{kg}$	Mass of shank	6.40
$l_{1} / \textrm{m}$	Length of thigh	0.394
$l_{2} / \textrm{m}$	Length of shank	0.516
$l_{c 1} / \textrm{m}$	Centers of masses of thigh	0.304
$J_{1} / \textrm{kg} \cdot \textrm{m}^{2}$	Moment of inertia of thigh	0.0590
$J_{2} / \textrm{kg} \cdot \textrm{m}^{2}$	Moment of inertia of shank	0.0175

We evaluate the tracking performance of our control approach and the other two control algorithms (i.e. fuzzy-PID and conventional MPC) both quantitatively and qualitatively. For quantitative description, the tracking performance is visualized in figures. Apparently, our controller show clear improves of tracking accuracy with disturbances.

For the quantitative evaluation, three performance indices, including the maximum tracking error $M_{e}$, the average $\mu$ and the standard deviation $\sigma$ of the tracking error, are utilized to evaluate the tracking accuracy of each method. They are defined as follows:

1. The maximum absolute value of the overall tracking errors, which is defined as,
  23 $$\begin{aligned} M_{e}=\max \{|e(i)|\} \quad i=1, \cdots , N \end{aligned}$$
  where *N* is the number of recorded samples. *e*(*i*) is the tracking error of each sample.
2. The average of the tracking error, which is defined as,
  24 $$\begin{aligned} \mu =\frac{1}{N} \sum _{1}^{N}|e(i)| \end{aligned}$$
3. The standard deviation of the tracking error, which is defined as,
  25 $$\begin{aligned} \sigma =\sqrt{\frac{1}{N} \sum _{1}^{N}(|e(i)|-\mu )^{2}} \end{aligned}$$

### Result and discussion

**Figure 4:**
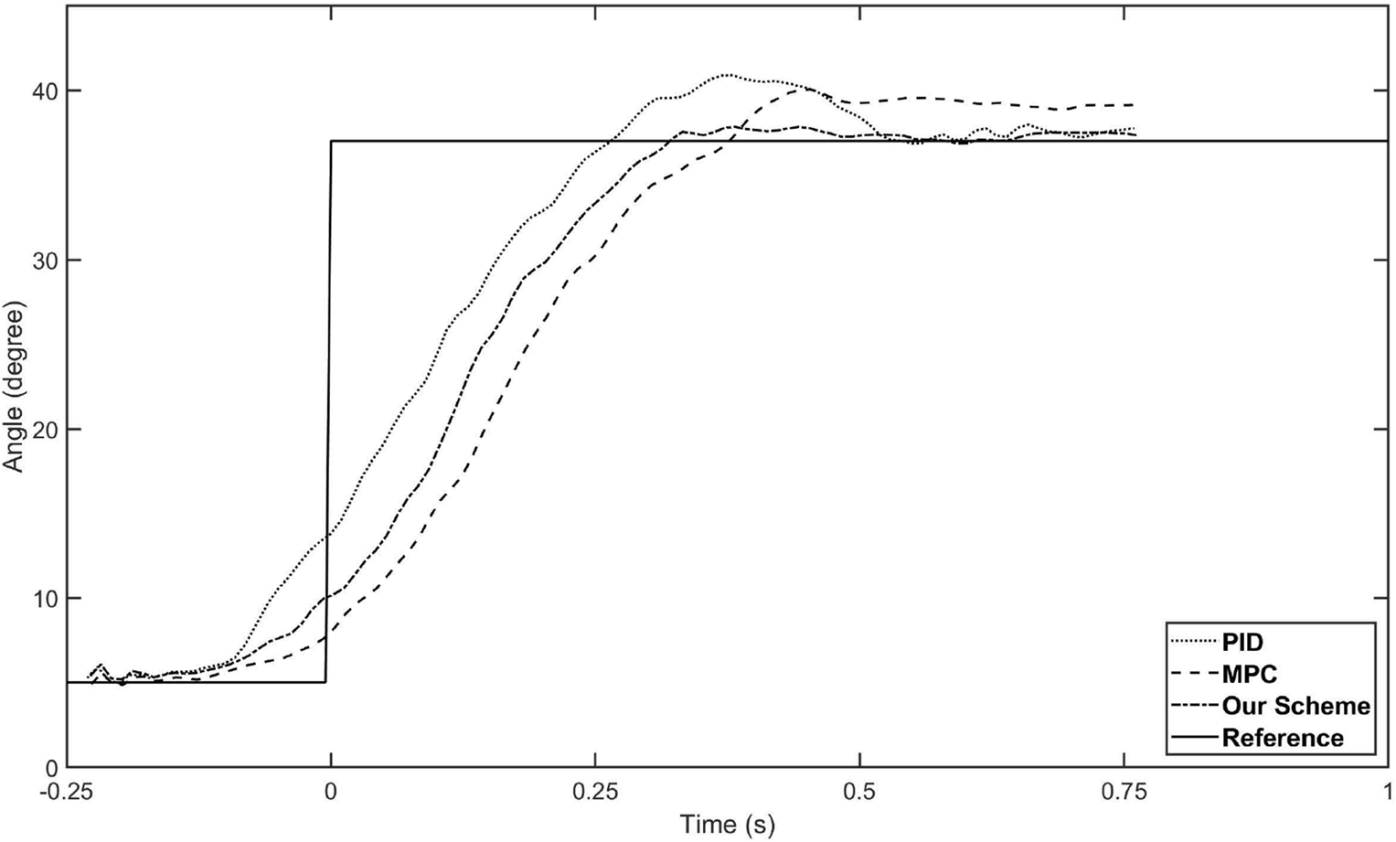
The tracking performance of three control scheme. The solid line is the reference trajectory we set as a step function. The dotted line represents the PID control result, the dashed line represents the MPC control result, and the dash-doted line represents our strategy control result.

Before formally tracking the trajectory of human lower limb, we first conducted a control test of hip joint angle to evaluate the speed of the proposed controller, and compared the result with fuzzy-PID and conventional MPC. The experimental conditions are consistent with the previous description, but the reference trajectory is set as a step function from 5 degrees to 37 degrees (as shown by the solid line in Fig. 4). The performances of the three controllers are shown in Fig. 4. It can be seen that our controller is inferior to PID in terms of control speed but superior to traditional MPC, and significantly superior to both in terms of overshoot. In addition, our strategy also has better performance in terms of terminal accuracy.

**Figure 5:**
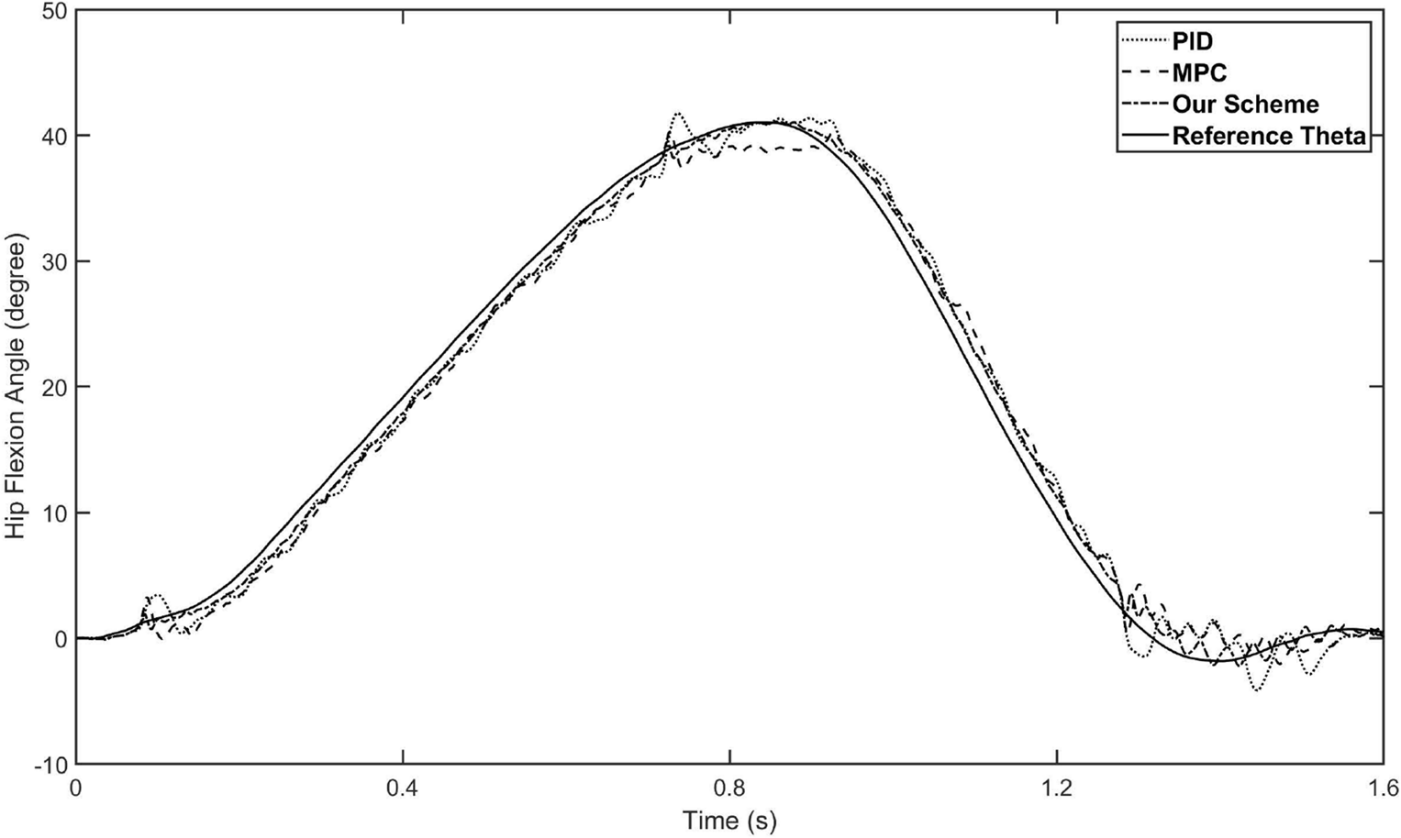
The tracking performance of three schemes for hip joint control. The solid line is the reference trajectory, which refers to the hip flexion angle of the human hip joint during one gait cycle. The dotted line represents the fuzzy-PID control result, the dashed line represents the conventional MPC control result, and the dash-doted line represents our strategy control result.

**Figure 6:**
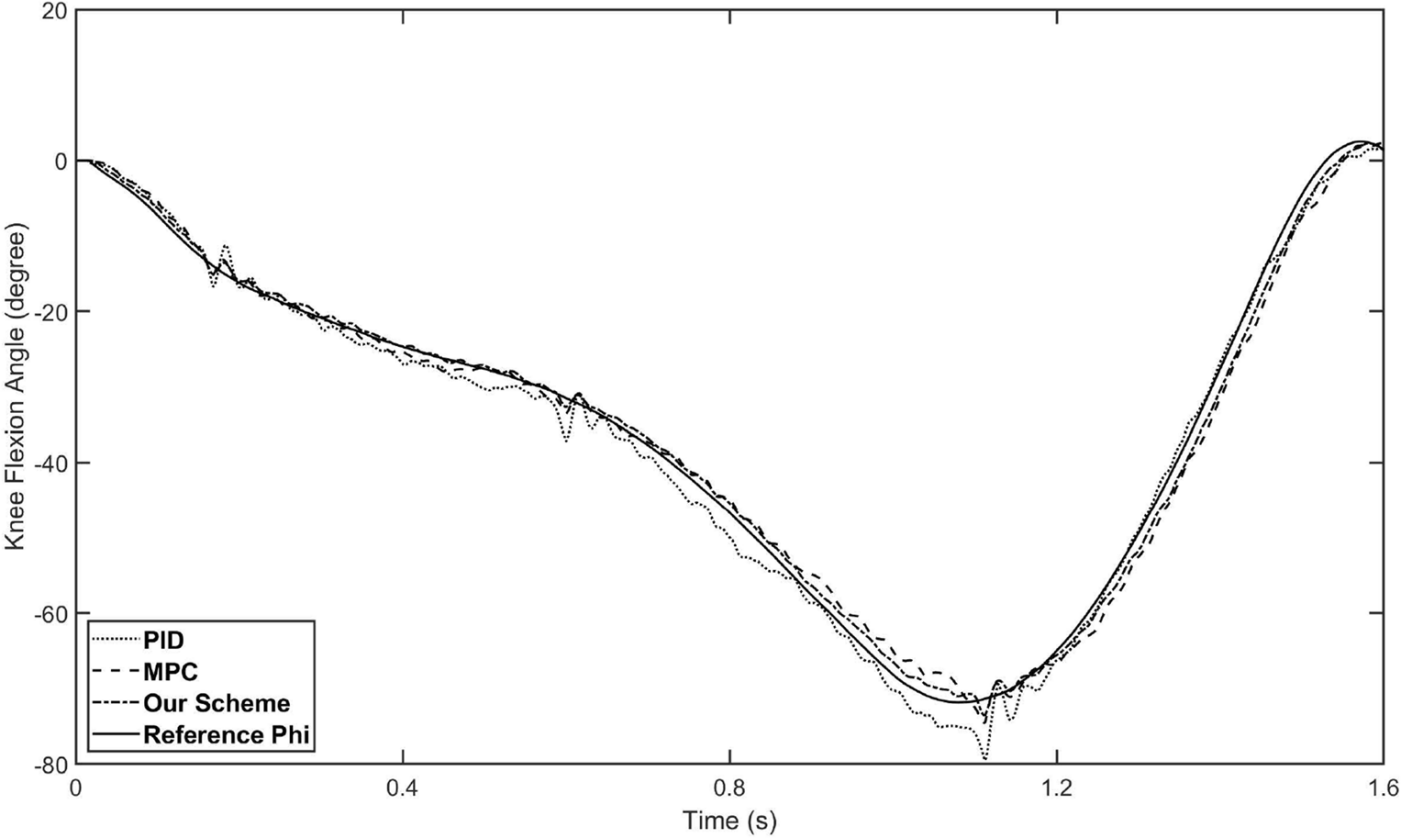
The tracking performance of three schemes for knee joint control. The solid line is the reference trajectory, which refers to the knee flexion angle of the human knee joint during one gait cycle. The dotted line represents the fuzzy-PID control result, the dashed line represents the conventional MPC control result, and the dash-doted line represents our strategy control result.

**Figure 7:**
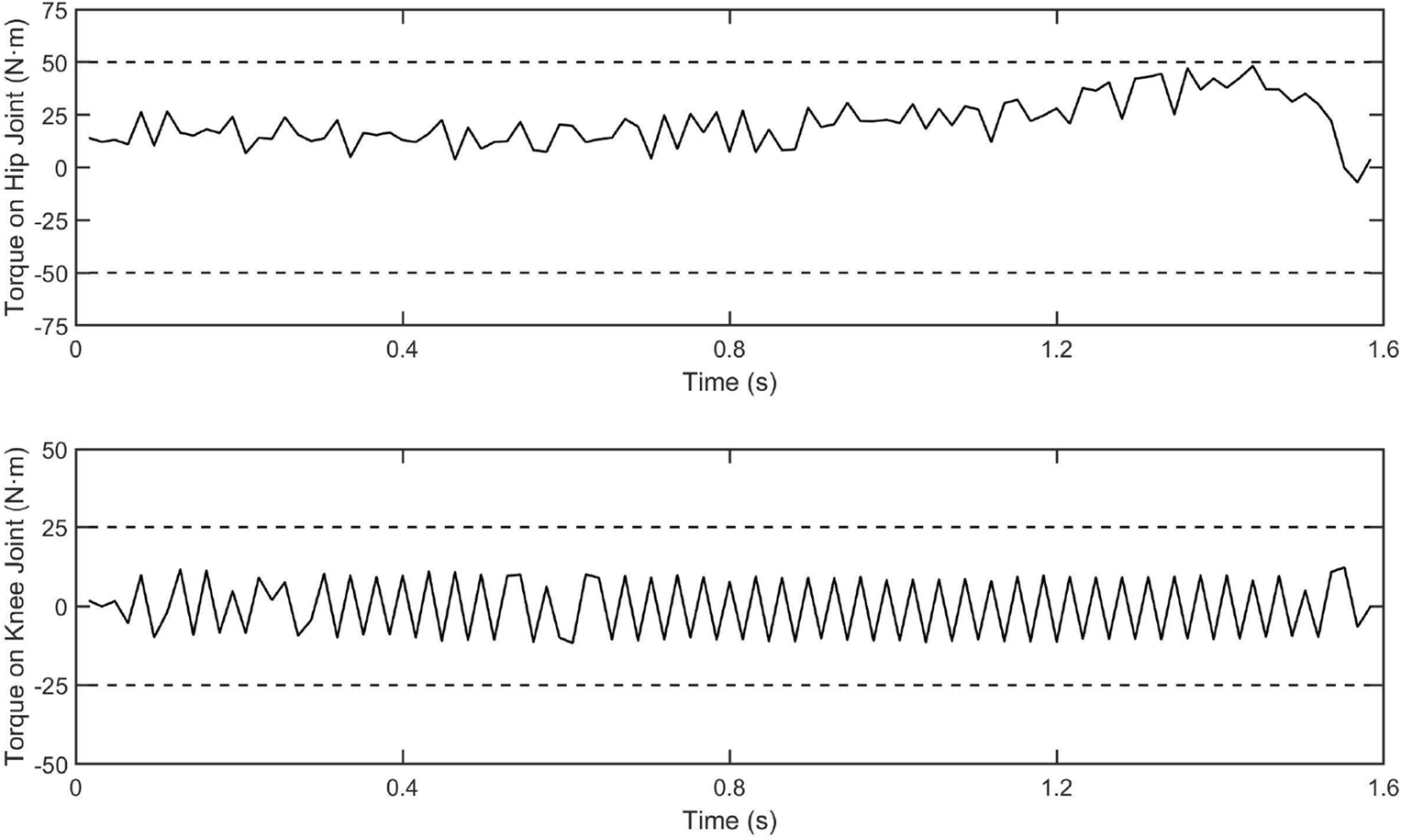
The control inputs of our control scheme. The upper sub-figure is the control torque on hip joint and the lower sub-figure is the control torque on knee joint.

The control input and the overall tracking performance of the aforementioned three controllers (i.e. fuzzy-PID, conventional MPC and our proposed controller) are shown in Figs. 5 and 6, respectively. The performance indices of the three controllers are collected in Table 2. The simulation results show that our proposed controller outperforms the other two controllers on tracking performance and has stronger robustness. With the help of the estimation by the designed ESO, our controller achieves higher tracking accuracy than conventional MPC controller, which also demonstrates the disturbances rejection ability of the designed ESO-based MPC controller. Specifically, the main reason why our controller surpasses conventional MPC is that it not only has the model-based compensation of MPC method, but also employed a linear ESO to estimate the total disturbance (in this case, external torques and modeling uncertainties), which is integrated to the output predictive equation of the system. In other words, by building a novel output prediction equation with ESO-estimated total disturbance, the response of the controlled exoskeleton system will be predicted more accurately, which enables the optimization solution of the control quantity to be obtained more precisely by solving the novel objective function containing disturbances. On the other hand, comparing Figs. 5 and 6, although the performance of conventional MPC controller is not as good as ours, it is better than fuzzy-PID, which indicates that the traditional MPC method still has acceptable robustness. Besides, the control inputs of the proposed controllers are bounded, as shown in the Fig. 7.

As shown in Table 2, our controller shows better tracking accuracy in all indices. By introducing linear ESO to the MPC, the mean tracking error of the proposed controller is reduced down to 0.93 degree (hip flexion angle) and 1.07 degree (knee flexion angle), while the mean tracking error of MPC controller is about 1.45 degree (hip flexion angle) and 1.61 degree (knee flexion angle). Compared with conventional MPC, the tracking performance of our controller is improved by about 34–35$\%$. Furthermore, our controller has greater advantage compared with fuzzy-PID, the tracking performance is improved by about 35–39$\%$.

**Table 2:** Performance indices of the aforementioned three control schemes.

Controller	Hip joint			Knee joint		
	$M_{e}$	$\mu$	$\sigma$	$M_{e}$	$\mu$	$\sigma$
Fuzzy-PID	3.9426	1.5380	1.7045	8.1416	1.6409	2.0806
Conventional MPC	3.2809	1.4357	1.6349	4.8485	1.6088	1.5904
Our scheme	2.1240	0.9279	1.0862	2.9393	1.0653	1.3122

## Conclusions

In this paper, we have proposed a disturbance rejection model predictive control strategy based on ESO for a lower limb rehabilitation exoskeleton. Our key is to integrate the estimation of system total disturbance to the quadratic objective function, which is used for solving the optimal control law of MPC. To do so, we first introduce virtual control variables to decouple the exoskeleton system, and then construct an ESO based on the expanded decoupled system, that can estimate the model uncertainties and the disturbance from the wearer as a total disturbance. Based on the stability theory of Lyapunov framework, the stability of the closed-loop system is ensured, which indicates that the proposed ESO-based MPC controller has prescribed tracking performance under the condition of disturbances and uncertainties. Through comparative virtual experimental results, we have demonstrated that our control scheme for exoskeleton outperforms two existing techniques (fuzzy-PID and conventional MPC) on the effectiveness and priority, in the strong disturbance case. As a result, our achievements will contribute to better rehabilitation training of patients using lower limb rehabilitation exoskeleton.

## Limitations and future works

In this paper, a certain simplification has been made to the physical model of the lower limb rehabilitation exoskeleton, and the control strategies have been proposed just for the gait in sagittal plane, to simplify the study. In addition, we regard the human-machine interaction forces and system parameter uncertainty as total disturbances to be observed and compensated, which poses poses a challenge to the performance of ESO. In future works, further refinement of the physical model of the exoskeleton can be carried out, and the problem of gait walking in three-dimensional space can be considered. In addition, the design of ESO can further observe interaction forces and uncertainties separately, depending on how the system is modeled.

## Data Availability

The datasets used and/or analysed during the current study available from the corresponding author on reasonable request.
